# Supplementary figures and images for: Autophagic flux is impaired in the brain tissue of Tay-Sachs disease mouse model
Source: PLoS One. 2023 Mar 16;18(3):e0280650. doi: 10.1371/journal.pone.0280650 (PMC10019743; doi:10.1371/journal.pone.0280650)

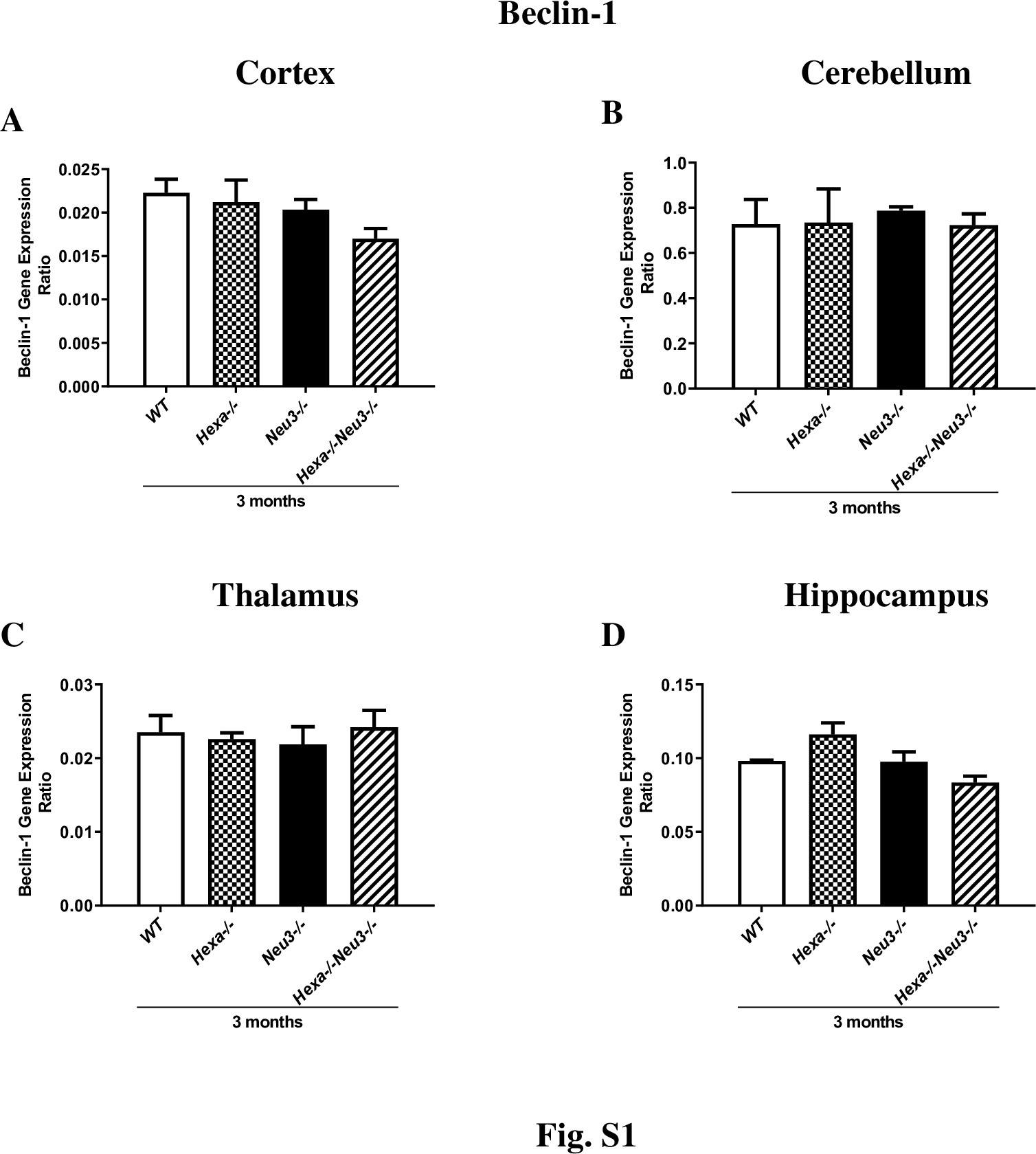

Supplement: S1 Fig — Beclin-1 gene expression levels of the cortex (A), cerebellum (B), thalamus (C), and hippocampus (D) of 3-month-old WT, Hexa-/-, Neu3-/-, Hexa-/-Neu3-/- mice. Expression ratios were calculated by the ΔCT method and percent ratios were represented. One-way ANOVA analysis was used to determine p-values via GraphPad. Data are reported as mean ± SEM (n = 2) (TIF) [file pone.0280650.s001.tif]

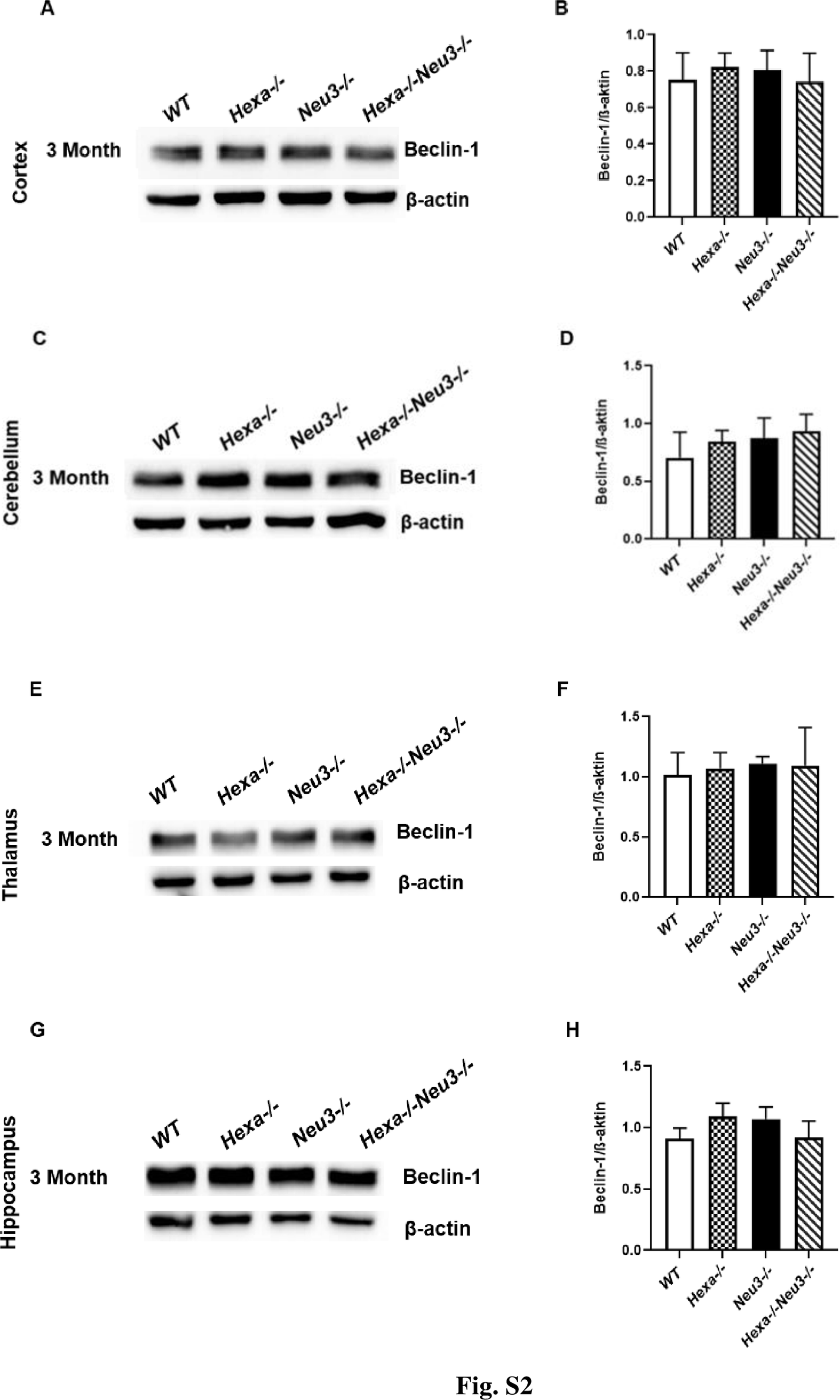

Supplement: S2 Fig — Immunoblot images and densitometric analysis of anti-Beclin-1 in the cortex (A, B), cerebellum (C, D), thalamus (E, F), and hippocampus (G, H) of 3-month-old WT, Hexa-/-, Neu3-/-, Hexa-/-Neu3-/- mice. β-actin as an internal control. Band intensities were determined by ImageJ and p values were determined by One-way-ANOVA analysis by GraphPad. Data are reported as mean ± SEM (n = 2) (TIF) [file pone.0280650.s002.tif]

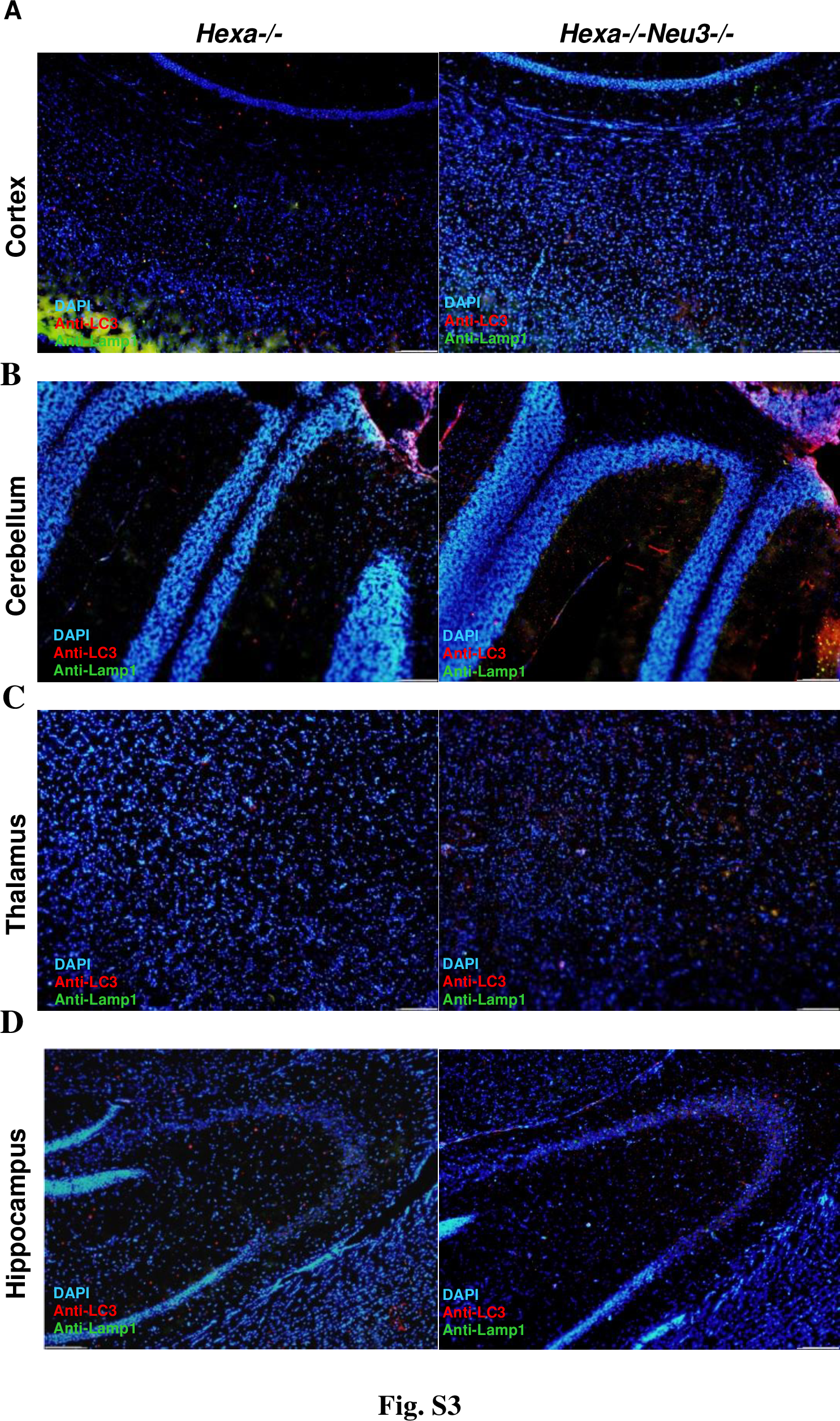

Supplement: S3 Fig — Immunohistochemical colocalization analysis images for cortex (A), cerebellum (B), thalamus (C), and hippocampus (D) sections from 2-months-old Hexa-/- and Hexa-/-Neu3-/- mice. The sections were stained with anti-LC3 antibody (red; Autophagosome marker), anti-Lamp1 (green; lysosomal marker), and DAPI (blue; nucleus). A yellow signal signifies the colocalization of LC3 and Lamp1 as autophagolysosome. Scale bar = 20 μm (TIF) [file pone.0280650.s003.tif]

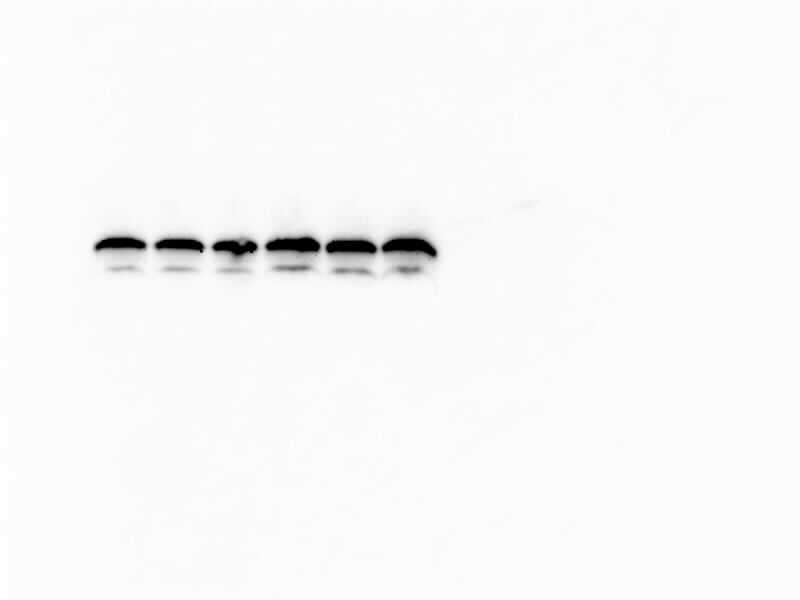

Supplement: S4 Fig — Immunohistochemical analysis images for cortex (A), cerebellum (B), thalamus (C), and hippocampus (D) sections from 2-months-old Hexa-/- and Hexa-/-Neu3-/- mice. The sections were stained with anti-p62 antibody (green; Autophagic termination marker) and DAPI (blue; nucleus). Scale bar = 20 μm (ZIP) [file pone.0280650.s004.zip › PACE Corrected/lc3 fihure 4j- 5months (first 4 bands, wt hexa neu3 hexaneu3.tif]

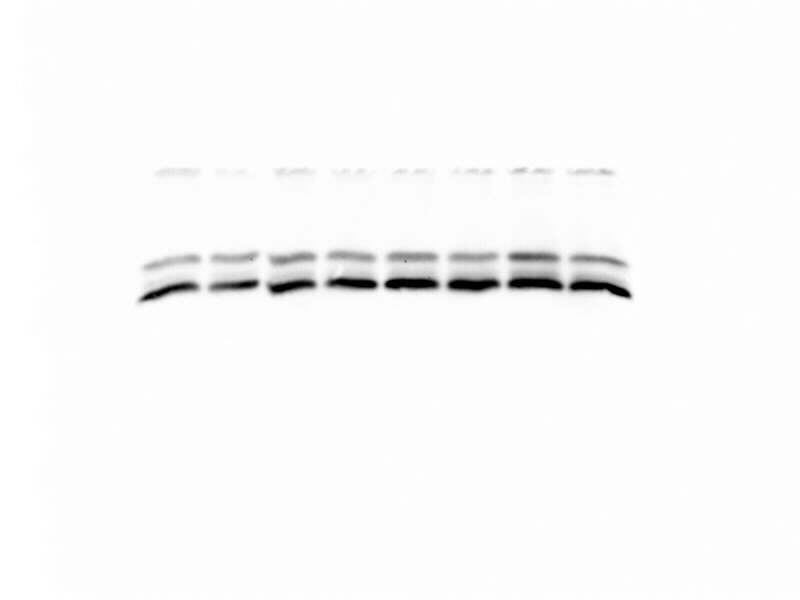

Supplement: S4 Fig — Immunohistochemical analysis images for cortex (A), cerebellum (B), thalamus (C), and hippocampus (D) sections from 2-months-old Hexa-/- and Hexa-/-Neu3-/- mice. The sections were stained with anti-p62 antibody (green; Autophagic termination marker) and DAPI (blue; nucleus). Scale bar = 20 μm (ZIP) [file pone.0280650.s004.zip › PACE Corrected/lc3 figure 4j- 2months (first 4 bands, wt hexa neu3 hexaneu3.tif]

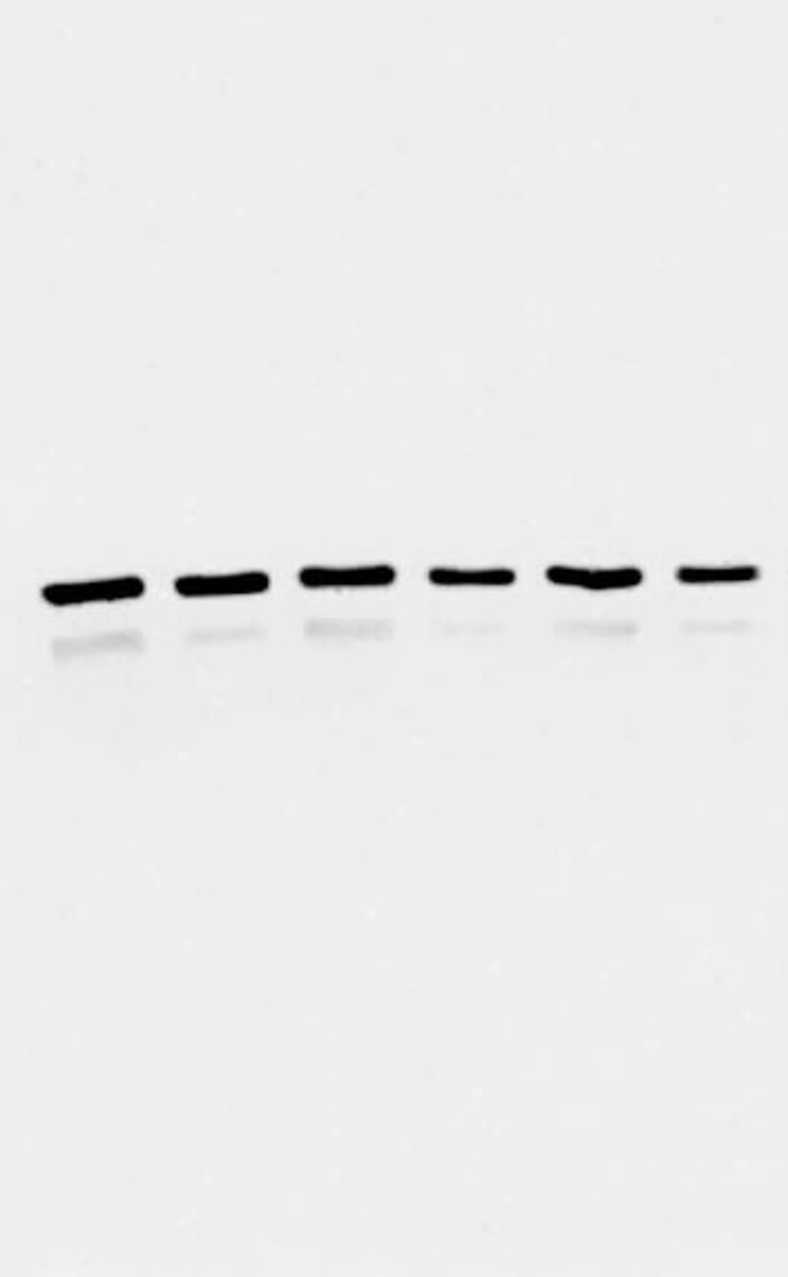

Supplement: S4 Fig — Immunohistochemical analysis images for cortex (A), cerebellum (B), thalamus (C), and hippocampus (D) sections from 2-months-old Hexa-/- and Hexa-/-Neu3-/- mice. The sections were stained with anti-p62 antibody (green; Autophagic termination marker) and DAPI (blue; nucleus). Scale bar = 20 μm (ZIP) [file pone.0280650.s004.zip › PACE Corrected/actin figure4j- 5months (first 4 bands, wt hexa neu3 hexaneu3.tif]

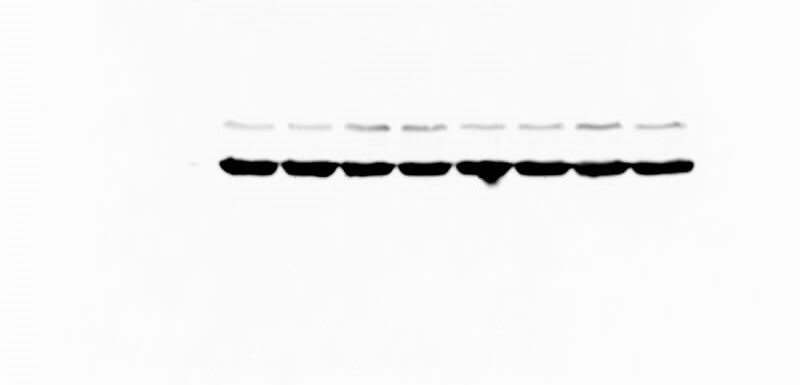

Supplement: S4 Fig — Immunohistochemical analysis images for cortex (A), cerebellum (B), thalamus (C), and hippocampus (D) sections from 2-months-old Hexa-/- and Hexa-/-Neu3-/- mice. The sections were stained with anti-p62 antibody (green; Autophagic termination marker) and DAPI (blue; nucleus). Scale bar = 20 μm (ZIP) [file pone.0280650.s004.zip › PACE Corrected/actin figure 4j- 2months ( first 4 bands, wt hexa neu3 hexaneu3.tif]

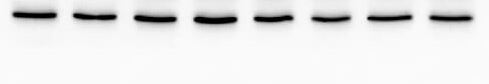

Supplement: S1 Raw data — (ZIP) [file pone.0280650.s005.zip › beclin1 p62 and LC3 raw data/cerebellum/bactin (2 and 5 months)/actin.JPG]

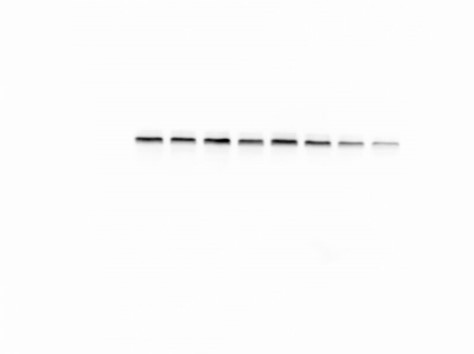

Supplement: S1 Raw data — (ZIP) [file pone.0280650.s005.zip › beclin1 p62 and LC3 raw data/cerebellum/beclin-1 2 and 5 months)/beclin1 cerebellum.jpg]

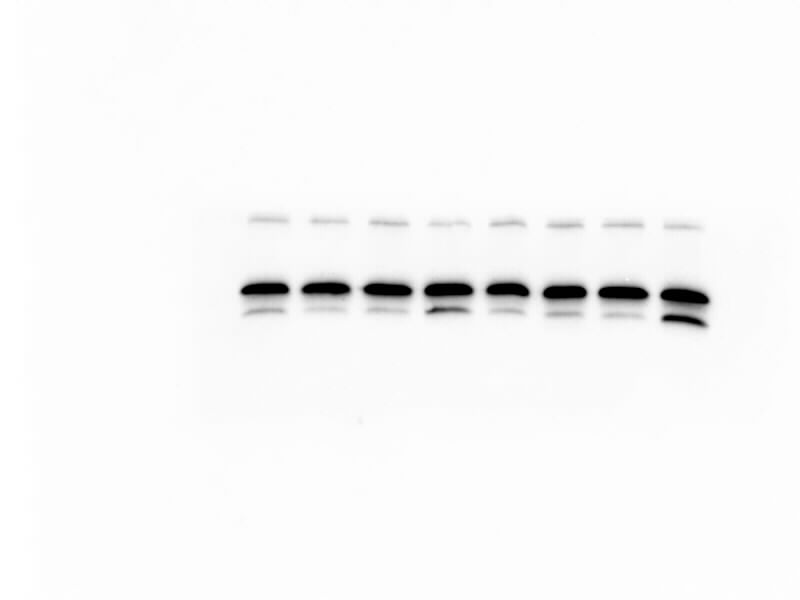

Supplement: S1 Raw data — (ZIP) [file pone.0280650.s005.zip › beclin1 p62 and LC3 raw data/cerebellum/lc3 (2 and 5 months)/lc3.JPG]

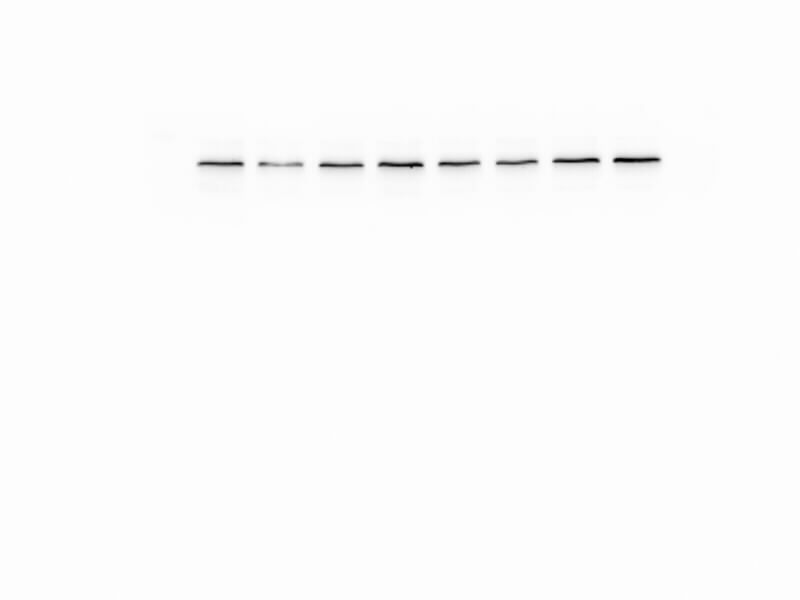

Supplement: S1 Raw data — (ZIP) [file pone.0280650.s005.zip › beclin1 p62 and LC3 raw data/cerebellum/p62 (2 and 5 months)/p62.JPG]

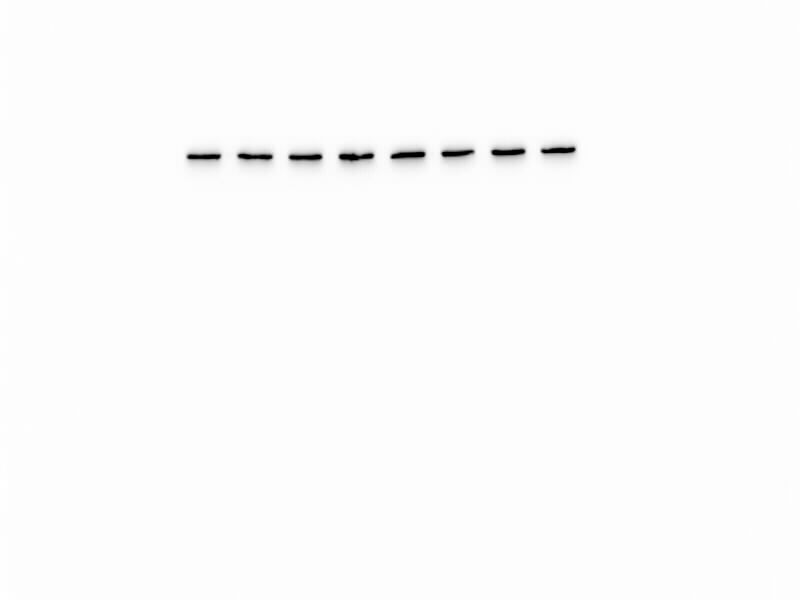

Supplement: S1 Raw data — (ZIP) [file pone.0280650.s005.zip › beclin1 p62 and LC3 raw data/cortex/actin (2and 5 months)/actin.JPG]

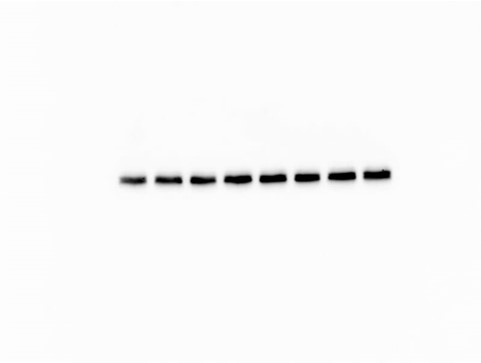

Supplement: S1 Raw data — (ZIP) [file pone.0280650.s005.zip › beclin1 p62 and LC3 raw data/cortex/Beclin1 (2 and 5 months)/beclin1 cortex.jpg]

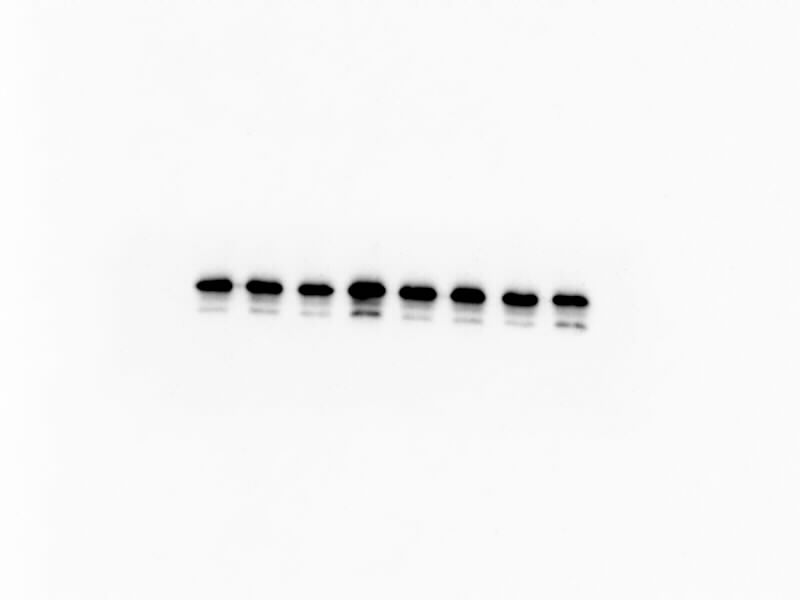

Supplement: S1 Raw data — (ZIP) [file pone.0280650.s005.zip › beclin1 p62 and LC3 raw data/cortex/lc3 (2 and 5months)/lc3.JPG]

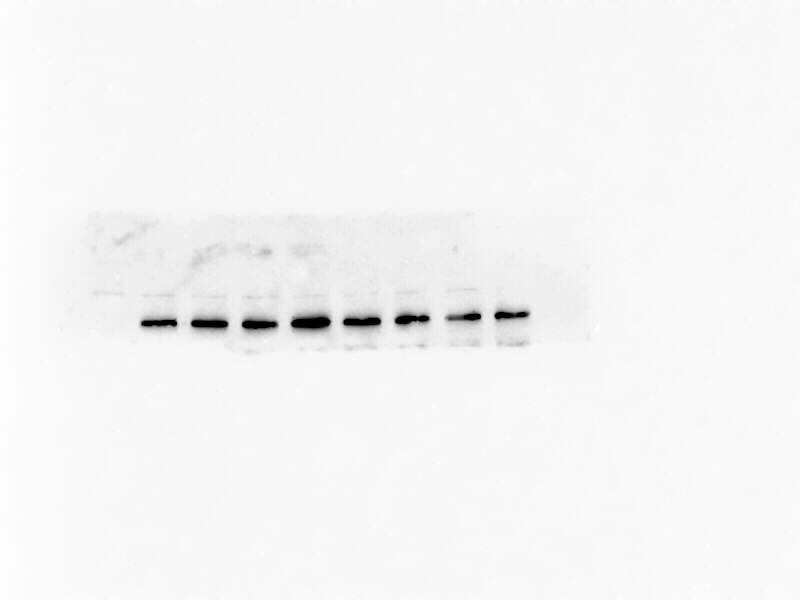

Supplement: S1 Raw data — (ZIP) [file pone.0280650.s005.zip › beclin1 p62 and LC3 raw data/cortex/p62 (2 and 5 months)/p62.JPG]

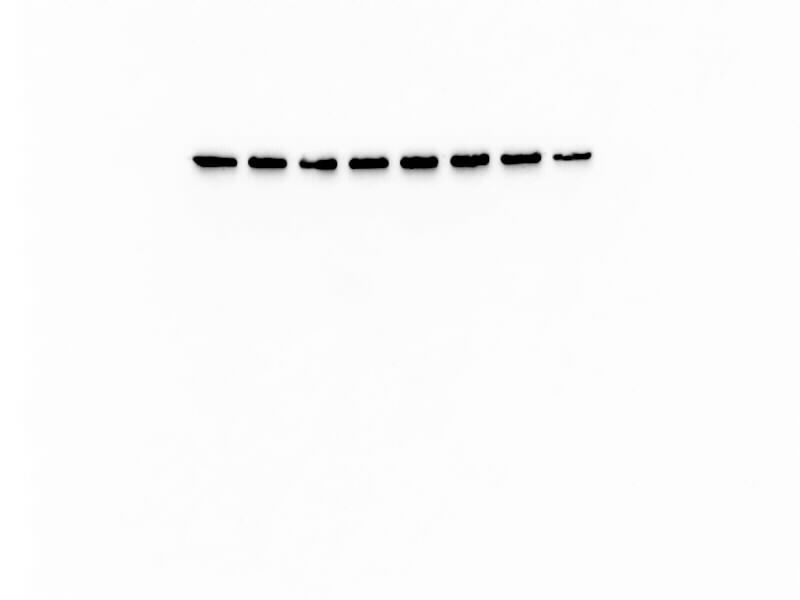

Supplement: S1 Raw data — (ZIP) [file pone.0280650.s005.zip › beclin1 p62 and LC3 raw data/hippocampus/actin (2 and 5 months)/actin.JPG]

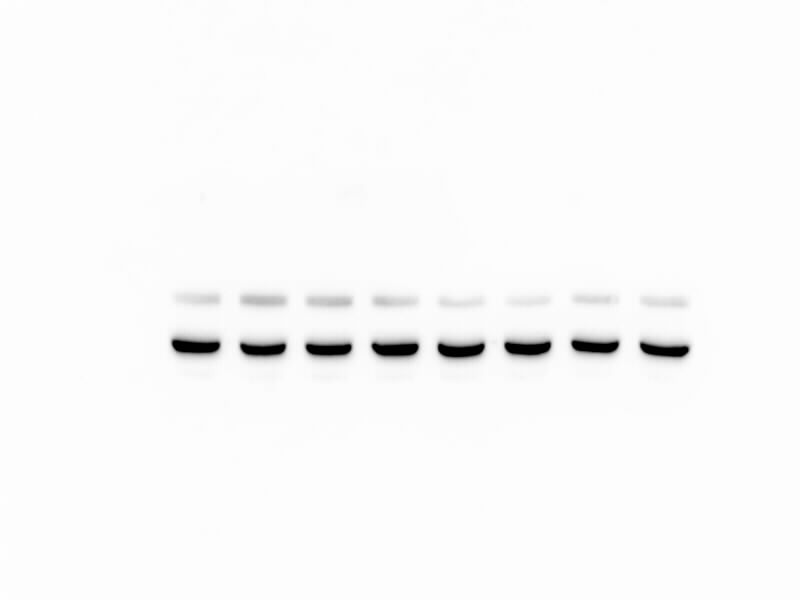

Supplement: S1 Raw data — (ZIP) [file pone.0280650.s005.zip › beclin1 p62 and LC3 raw data/hippocampus/beclin1 (2and 5 months)/actin.JPG]

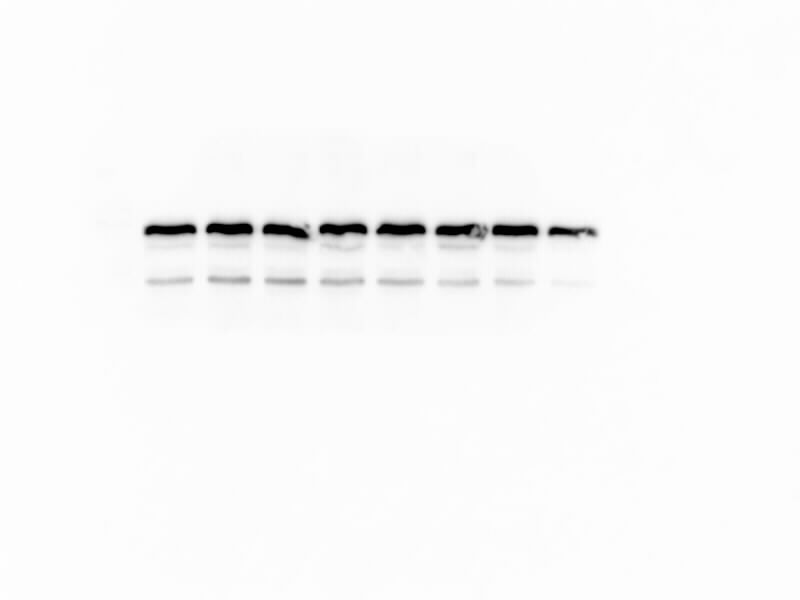

Supplement: S1 Raw data — (ZIP) [file pone.0280650.s005.zip › beclin1 p62 and LC3 raw data/hippocampus/beclin1 (2and 5 months)/beclin1.JPG]

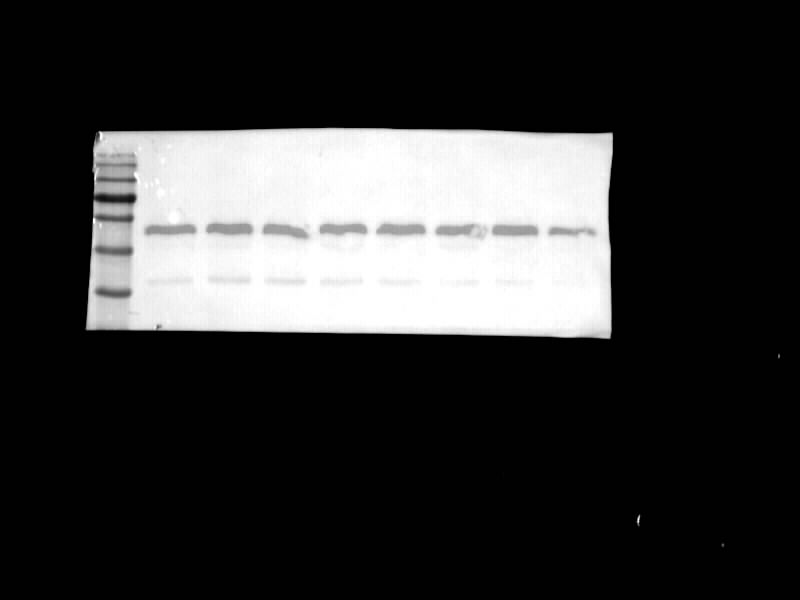

Supplement: S1 Raw data — (ZIP) [file pone.0280650.s005.zip › beclin1 p62 and LC3 raw data/hippocampus/beclin1 (2and 5 months)/merged beclin1.JPG]

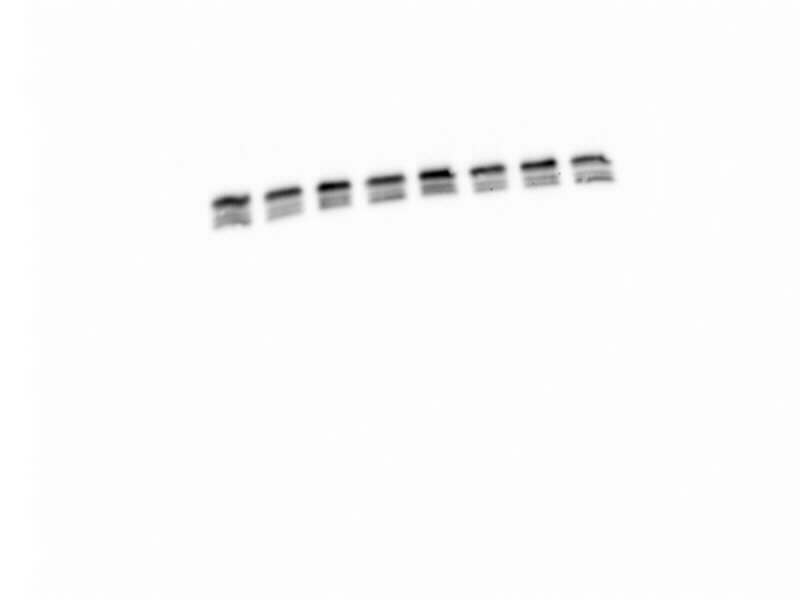

Supplement: S1 Raw data — (ZIP) [file pone.0280650.s005.zip › beclin1 p62 and LC3 raw data/hippocampus/lc3 (2 and 5 months)/lc3.JPG]

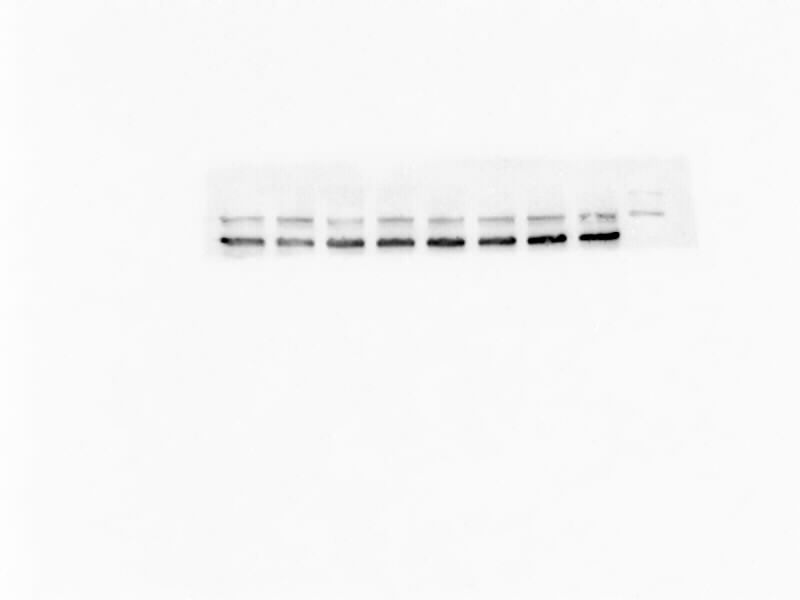

Supplement: S1 Raw data — (ZIP) [file pone.0280650.s005.zip › beclin1 p62 and LC3 raw data/hippocampus/p62 (2 and 5months)/p62.JPG]

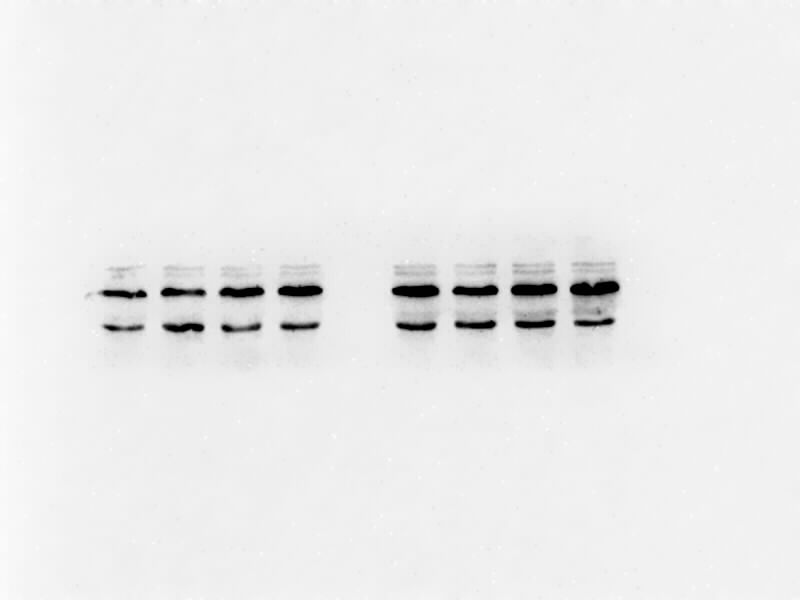

Supplement: S1 Raw data — (ZIP) [file pone.0280650.s005.zip › beclin1 p62 and LC3 raw data/thalamus/actin (2 and 5 months)/actin (after p62, actin is observed).JPG]

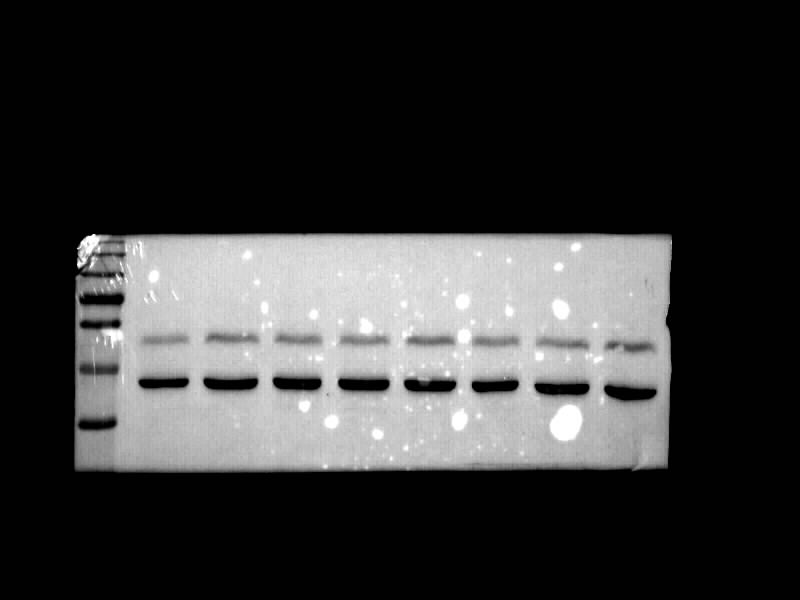

Supplement: S1 Raw data — (ZIP) [file pone.0280650.s005.zip › beclin1 p62 and LC3 raw data/thalamus/beclin1 (2 and 5 months)/actin/merged.JPG]

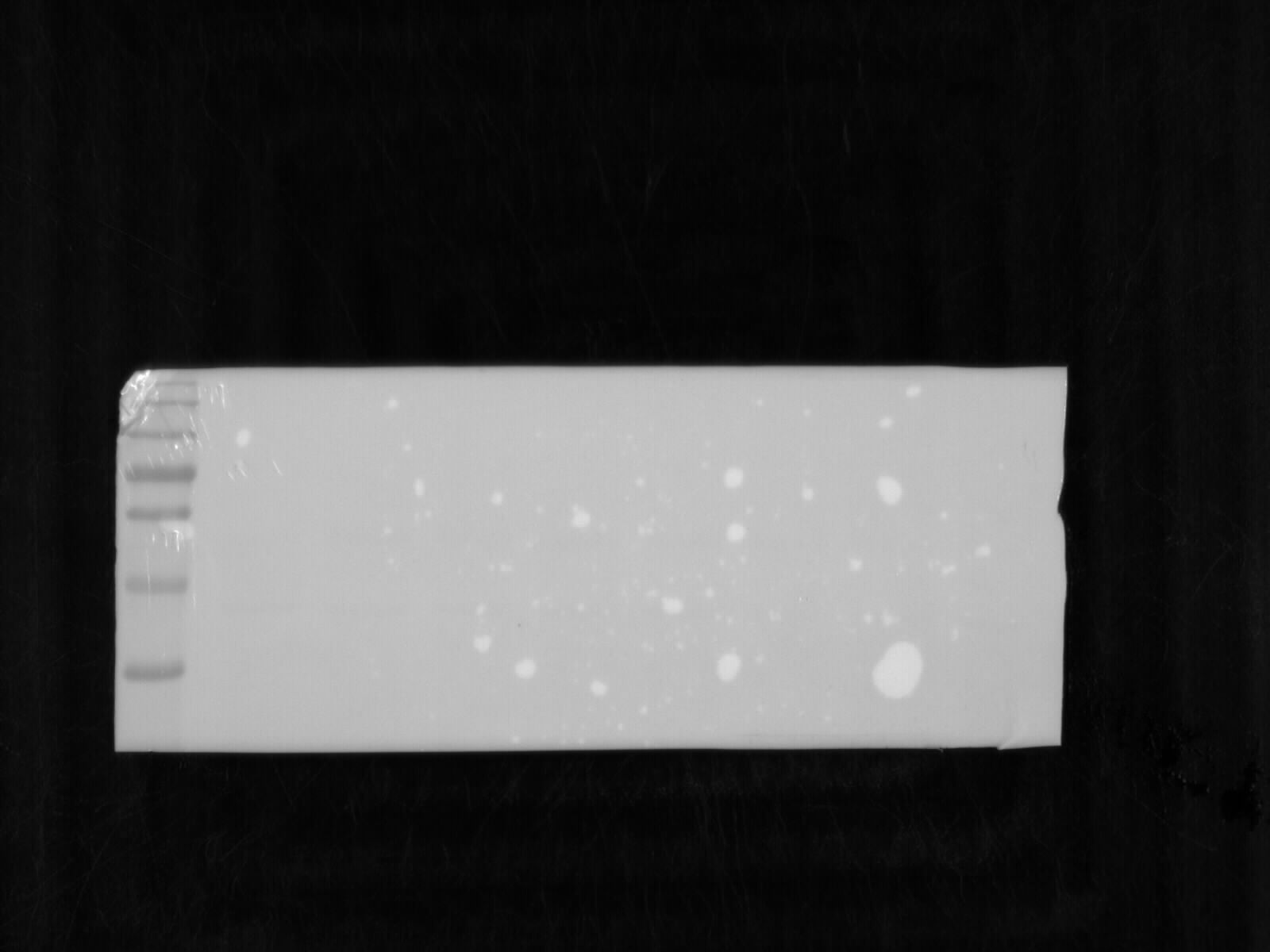

Supplement: S1 Raw data — (ZIP) [file pone.0280650.s005.zip › beclin1 p62 and LC3 raw data/thalamus/beclin1 (2 and 5 months)/actin/mmarkerr.JPG]

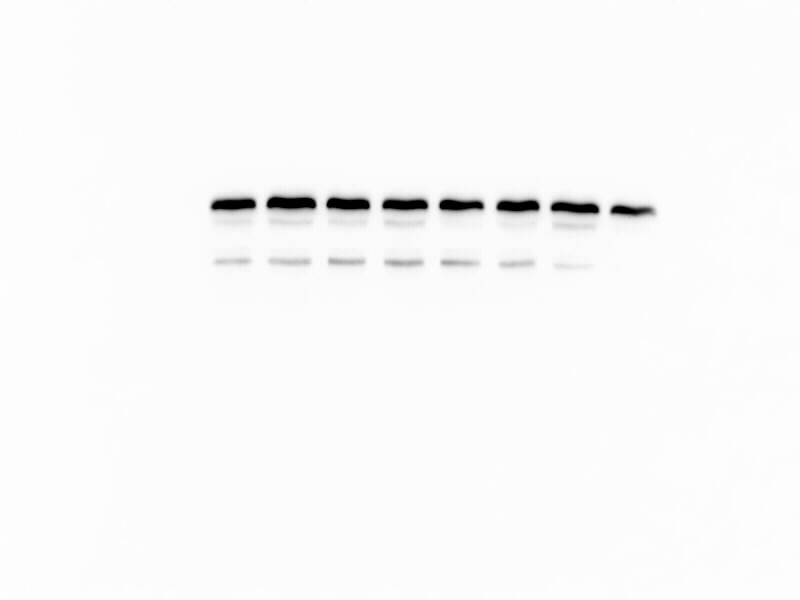

Supplement: S1 Raw data — (ZIP) [file pone.0280650.s005.zip › beclin1 p62 and LC3 raw data/thalamus/beclin1 (2 and 5 months)/beclin-1/beclin1.JPG]

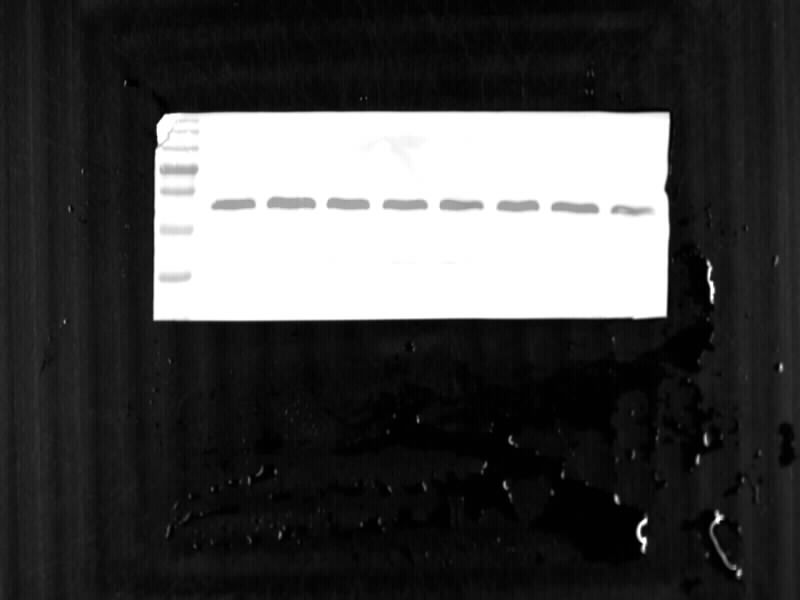

Supplement: S1 Raw data — (ZIP) [file pone.0280650.s005.zip › beclin1 p62 and LC3 raw data/thalamus/beclin1 (2 and 5 months)/beclin-1/merged.JPG]

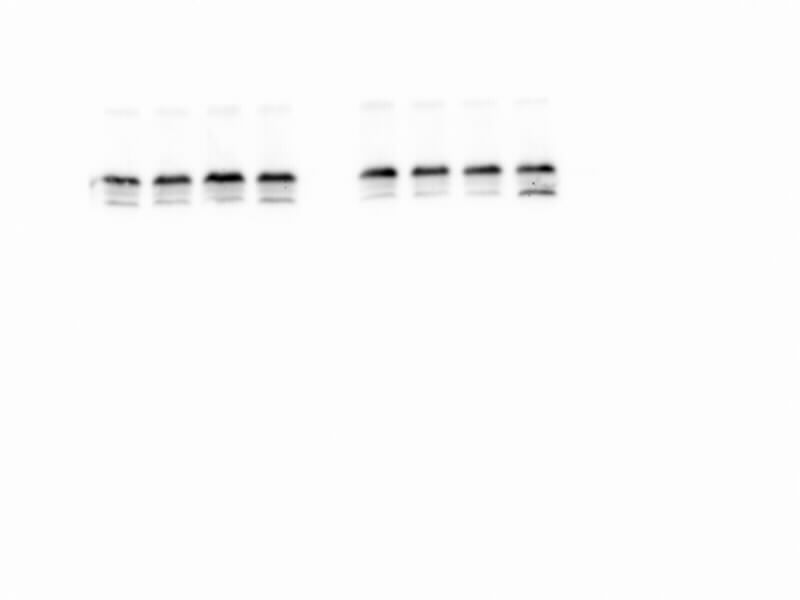

Supplement: S1 Raw data — (ZIP) [file pone.0280650.s005.zip › beclin1 p62 and LC3 raw data/thalamus/lc3 (2 and 5 months)/lc3.JPG]

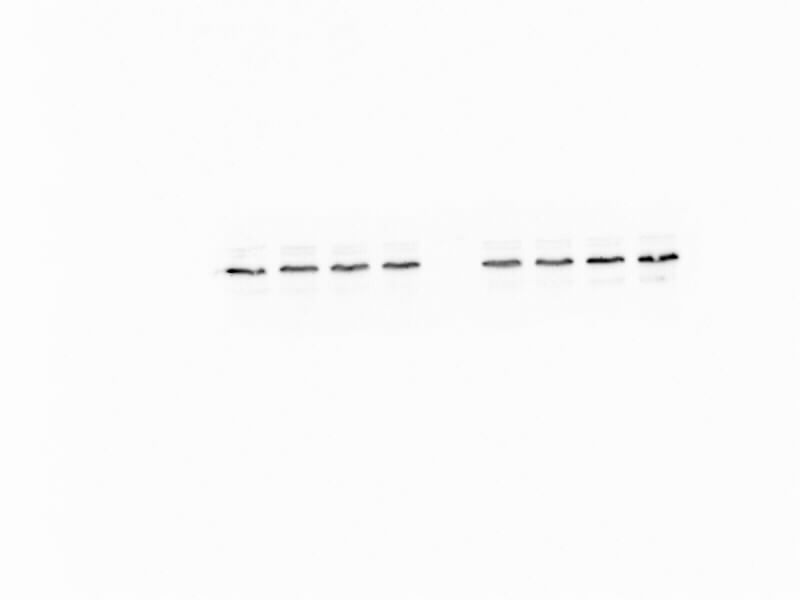

Supplement: S1 Raw data — (ZIP) [file pone.0280650.s005.zip › beclin1 p62 and LC3 raw data/thalamus/p62 (2 and 5months)/p62.JPG]
