# Supplementary material for: Autophagic flux is impaired in the brain tissue of Tay-Sachs disease mouse model
Source: PLoS One. 2023 Mar 16;18(3):e0280650. doi: 10.1371/journal.pone.0280650 (PMC10019743; doi:10.1371/journal.pone.0280650)
Supplement: Attachment — Submitted filename: PONE Response to reviewers.docx [file pone.0280650.s006.docx]

**Response to reviewers**

Reviewer 1:

This study aims to investigate alterations in autophagic flux in the new mouse model of Tay-Sachs disease, which should provide information for future mechanistic and therapeutic studies in this field.

1. However, this study only includes 3 mice each groups, lacking statistical rigor.

**All data presented here were replicated in at least three mice with all histochemical staining data quantified by experimenters who were blind to genotypes/treatments. Animals were randomly allocated into control and experimental groups.**

2. The implications of alterations of those gene expression are not **well-explained**.

The rationale why the authors are looking at a certain gene is not fully explained.

**The rationale for why looking at a certain gene expression is explained in the introduction section of the manuscript as below**

**In mammalian cells, two major mechanisms are carried out for the degradation of intracellular proteins which are the ubiquitin-proteasome system (UPS) and autophagy [6]. In particular, autophagy is involved in lysosome-dependent pathways for damaged organelles, unfolded proteins, and accumulated cellular components to maintain cellular homeostasis [7]. Autophagic flux includes vesicle trafficking and a network in which newly produced autophagosomes (double-membrane vesicles) are fused with lysosomes to degrade autophagic cargo. In this process, autophagore formation, autophagosome completion with the closure of membrane, and autophagosome-lysosome fusion (autolysosome) take place respectively [8]. Each step of autophagic flux is finely regulated by specific protein complexes and the initiation step, the sequestering of autophagic cargo within an isolation membrane (phagophore), is controlled by the Beclin-1. Beclin-1 consists of three distinct structural domains interacting with proteins that regulate autophagy machinery. In particular, Beclin-1, which can interact with several cofactors, promotes the formation of the Beclin-1-Vps34-Vps15 core body, in this way induces the initiation of autophagic flux [9]. Autophagy-related (Atg) proteins generate phagophore assembly sites and enable the envelopment of cytoplasmic material. In particular, the Atg9 protein is a key regulator of autophagy induction. During the maturation of autophagosome, Atg7 has involved the conversion of the cytosolic form of microtubule-associated protein 1 light chain 3 (LC3-I) to LC3-II which is located on both inner and outer autophagosomal membranes [9]. LC3 is involved in phagophore edge folding that resulted in autophagosome formation. After the formation of autolysosomes formed by the combination of autophagosomes and lysosomes, LC3-II on the outer membrane is converted back to LC3-I and then intra-autophagosomal LC3-II is degraded by lysosomal hydrolyses. Therefore, the level of LC3-II as a marker of dynamic autophagosomal membranes is generally studied to monitor autophagic activity [10]. In addition, the level of ubiquitin-binding scaffold protein p62 (aggregated endogenous substrates) which know to be associated with LC3-II in the autophagosome is mostly evaluated as a marker of termination of autophagy [9,11]. A bunch of studies also showed a defect in autophagic flux and secondary accumulation of autophagic substrates such as autophagosomes in several LSDs [6,12–14].**

It is difficult to follow why change of one gene in a certain brain region means something for a better understanding of pathogenesis mechanisms of this mouse model.

Moreover, how the information collected in this study will contribute to a better understanding of Tay-Sachs disease and this mouse model is not clear.

Basically, changes of several genes in some brain regions (but not others) at a certain time is only an indicator (weak evidence) of something.

This study lacks a clear logic, strong evidence, and a meaningful conclusion.

**In this study, we show that abnormal GM2 accumulation in the different region of brain tissue from TSD mouse model leads to impaired autophagy evident from attenuated expression of some components of the autophagy network, decreased autophagosome formation, and reduced autophagy flux. We for the first time study the expression of some fundamental autophagic markers (LC3, p62, and Beclin-1) in a TSD murine model by q-RT-PCR, immunohistochemistry and Western blot.**

3. The reviewer is not convinced that targeting autophagic flux could be potentially therapeutic, which seems to be a conclusion of this study.

**We are sorry for not convincing the reviewer that targeting autophagic flux could be potentially therapeutic in this manuscript. However, we have some preliminary in vitro data indicating lithium as a therapeutic agent could be used to restore dysregulated autophagy in Tay-Sachs cells. That data is not completed yet to add to the manuscript.**

**Although additional studies are required to fully characterize the autophagy machinery in the recently generated TSD murine model, this paper suggests impaired autophagy as a new aspect involved in the molecular pathogenesis of TSD. Despite important advances made in the last years in TSD research, the principally studied therapies such as gene and anti-inflammatory therapy and their combination can increase the life expectancy of the treated TSD mice (unpublished data yet) autophagy modulation could be a promising option to be tested in combination therapies to help to achieve a complete TSD phenotype rescue.**

4. There are many grammar mistakes.

**In this final version of the manuscript, we used a free online writing assistant called Grammarly**

Reviewer 2:

The authors examined autophagic flux initiation, autophagosome number in the elongation, and p62/SQSTM1 in termination in 2-month and 5-month TSD mice model cortex, cerebellum, thalamus and hippocampus by qRT-PCR, Western Blot, and immunohistochemical techniques.

The data revealed accumulated autophagosomes, indicating impairment in autophagic flux in the brain.

This work elucidates the contribution of altered autophagy to TSD pathology. I would like to see the following points addressed in the manuscript before its publication.

1. Hexa-/-Neu3-/- undergo progressive neurodegeneration and survived up to 5 months. However, no significant difference in the protein level of Beclin-1 was detected in 2-month-old WT, Hexa-/-, Neu3-/-, and Hexa-/-Neu3-/- mice. Thus, more time point data is necessary to determine when autophagic flux alteration in those gene KO mice.

**We added one more time point (3-months old mice) to determine when autophagic flux alterations (Fig Supp 1 and Fig Supp 2)**

2. LC3 protein level in cortex and cerebellum regions of 2-month-old Hexa-/-Neu3-/- mice was notably elevated compared to WT. It is necessary to analyze the number of LC3(+) vesicles in cortex and cerebellum using immunohistochemistry.

**We added new data showing LC3 protein levels in cortex and cerebellum regions of 2-month-old mice using immunohistochemistry (Fig Supp 3)**

3. P62 protein level was significantly elevated shown in both cortex and hippocampus of 2-month-old Hexa-/-Neu3-/- mice compared to WT. It is necessary to analyze the P62 in the cortex and hippocampus using immunohistochemistry.

**We added new data showing p62 protein levels in cortex and cerebellum regions of 2-month-old mice using immunohistochemistry (Fig Supp 4)**
